# Neo-epitope detection identifies extracellular matrix turnover in systemic inflammation and sepsis: an exploratory study

**DOI:** 10.1186/s13054-024-04904-4

**Published:** 2024-04-12

**Authors:** YiWen Fan, Jill Moser, Matijs van Meurs, Dorien Kiers, Jannie Marie Bülow Sand, Diana Julie Leeming, Peter Pickkers, Janette K. Burgess, Matthijs Kox, Janesh Pillay

**Affiliations:** 1 Department of Pathology and Medical Biology, University Medical Center Groningen, University of Groningen, Groningen, The Netherlands; 2 Department of Critical Care, University Medical Center Groningen, University of Groningen, Hanzeplein 1, 9713 GZ Groningen, The Netherlands; 3 University Medical Center Groningen, Research Institute for Asthma and COPD, University of Groningen, Groningen, The Netherlands; 4 Department of Intensive Care Medicine, Radboud University Medical Center, Nijmegen, The Netherlands; 5 Nordic Bioscience, Hepatic and Pulmonary Research, Herlev, Denmark; 6 Radboud Centre for Infectious Diseases (RCI), Radboud University Medical Center, Nijmegen, The Netherlands

**Keywords:** Extracellular matrix turnover, Collagen, Neo-epitope, Human endotoxemia, Sepsis

## Abstract

**Background:**

Sepsis is associated with high morbidity and mortality, primarily due to systemic inflammation-induced tissue damage, resulting organ failure, and impaired recovery. Regulated extracellular matrix (ECM) turnover is crucial for maintaining tissue homeostasis in health and in response to disease-related changes in the tissue microenvironment. Conversely, uncontrolled turnover can contribute to tissue damage. Systemic Inflammation is implicated to play a role in the regulation of ECM turnover, but the relationship between the two is largely unclear.

**Methods:**

We performed an exploratory study in 10 healthy male volunteers who were intravenously challenged with 2 ng/kg lipopolysaccharide (LPS, derived from *Escherichia coli*) to induce systemic inflammation. Plasma samples were collected before (T0) and after (T 1 h, 3 h, 6 h and 24 h) the LPS challenge. Furthermore, plasma was collected from 43 patients with septic shock on day 1 of ICU admission. Circulating neo-epitopes of extracellular matrix turnover, including ECM degradation neo-epitopes of collagen type I (C1M), type III (C3M), type IV (C4Ma3), and type VI (C6M), elastin (ELP-3) and fibrin (X-FIB), as well as the ECM synthesis neo-epitopes of collagen type III (PRO-C3), collagen type IV (PRO-C4) and collagen type VI (PRO-C6) were measured by ELISA. Patient outcome data were obtained from electronic patient records.

**Results:**

Twenty-four hours after LPS administration, all measured ECM turnover neo-epitopes, except ELP-3, were increased compared to baseline levels. In septic shock patients, concentrations of all measured ECM neo-epitopes were higher compared to healthy controls. In addition, concentrations of C6M, ELP-3 and X-FIB were higher in patients with septic shock who ultimately did not survive (N = 7) compared to those who recovered (N = 36).

**Conclusion:**

ECM turnover is induced in a model of systemic inflammation in healthy volunteers and was observed in patients with septic shock. Understanding interactions between systemic inflammation and ECM turnover may provide further insight into mechanisms underlying acute and persistent organ failure in sepsis.

**Supplementary Information:**

The online version contains supplementary material available at 10.1186/s13054-024-04904-4.

## Background

Sepsis, caused by the dysregulated host response to infection, is characterized by systemic inflammation and organ failure, and is associated with high morbidity and mortality [1, 2]. Tissue injury, resulting organ failure, and impaired recovery drives this mortality. Excessive and dysregulated inflammation has been proposed to play a major role in initiating tissue injury. Therefore, until now, research has mainly focused on the role of inflammation in these syndromes. Little attention has been paid to the turnover and remodelling of the extracellular matrix (ECM), a crucial component of tissue integrity and function, and pivotal to tissue repair and organ recovery [3].

Tissue damage is accompanied by disruption of the ECM, a network of fibrous and other proteins and glycosaminoglycans present in all organs that provide biomechanical and biochemical cues to cells [4]. Normal ECM composition and architecture are crucial for the maintenance of tissue structure and function [5]. The ECM is dynamic, with regulated turnover of the ECM being important for tissue homeostasis [3, 6]. ECM remodelling involves the synthesis, degradation, and other post-translational modifications of ECM components, which are essential for microenvironment homeostasis and tissue repair during wound healing [3]. During ECM remodelling, neo-epitopes from ECM proteins can be released into the circulation [7]. These neo-epitopes are generated when proteases, such as matrix-metalloproteinases (MMP) and a disintegrin and metalloproteinase with thrombospondin motifs (ADAMTS), either release pro-peptides from ECM fibres as part of the extracellular assembly of newly forming ECM fibrils, or when the proteases cleave fragments from ECM proteins residing within tissues. The fragments are generated in a regulated manner, with proteases cleaving ECM proteins at defined amino acid sites within the protein structures, thereby exposing neo-epitopes on the ECM fragments that are released into the circulation.

Circulating ECM neo-epitopes could potentially serve as easily measurable non-invasive tests indicative of disease status and activity. In many chronic inflammatory diseases, neo-epitopes of several different circulating collagens including C1M, C3M, C6M have been shown to reflect and predict disease severity and progression [8–10]. However, in acute inflammatory syndromes, such as sepsis, the potential of circulating ECM neo-epitopes as indicators of tissue remodelling during organ failure and repair has not been extensively explored. In patients suffering from these syndromes, increases in collagen fragments reflecting degradation and synthesis in the circulation and bronchoalveolar lavage fluid have been reported, although these studies have mainly focused on collagen I and III [11, 12]. In contrast, several studies have reported impaired collagen synthesis in sepsis, through measurement of procollagens type I and III [13, 14].

In this exploratory study, we examined the presence of a defined circulating ECM signature following an inflammatory insult in humans and the potential use of ECM neo-epitopes as biomarkers of disease severity in sepsis. Therefore, we firstly explored if systemic inflammation alone was sufficient to induce ECM turnover in a human model of systemic inflammation induced by intravenous administration of bacterial lipopolysaccharide (LPS, also known as the experimental human endotoxemia model). Thereafter, we quantified circulating ECM neo-epitopes in a cohort of patients with septic shock admitted to the Intensive Care Unit.

## Materials and methods

### Experimental human endotoxemia subjects

**Fig. 1:**
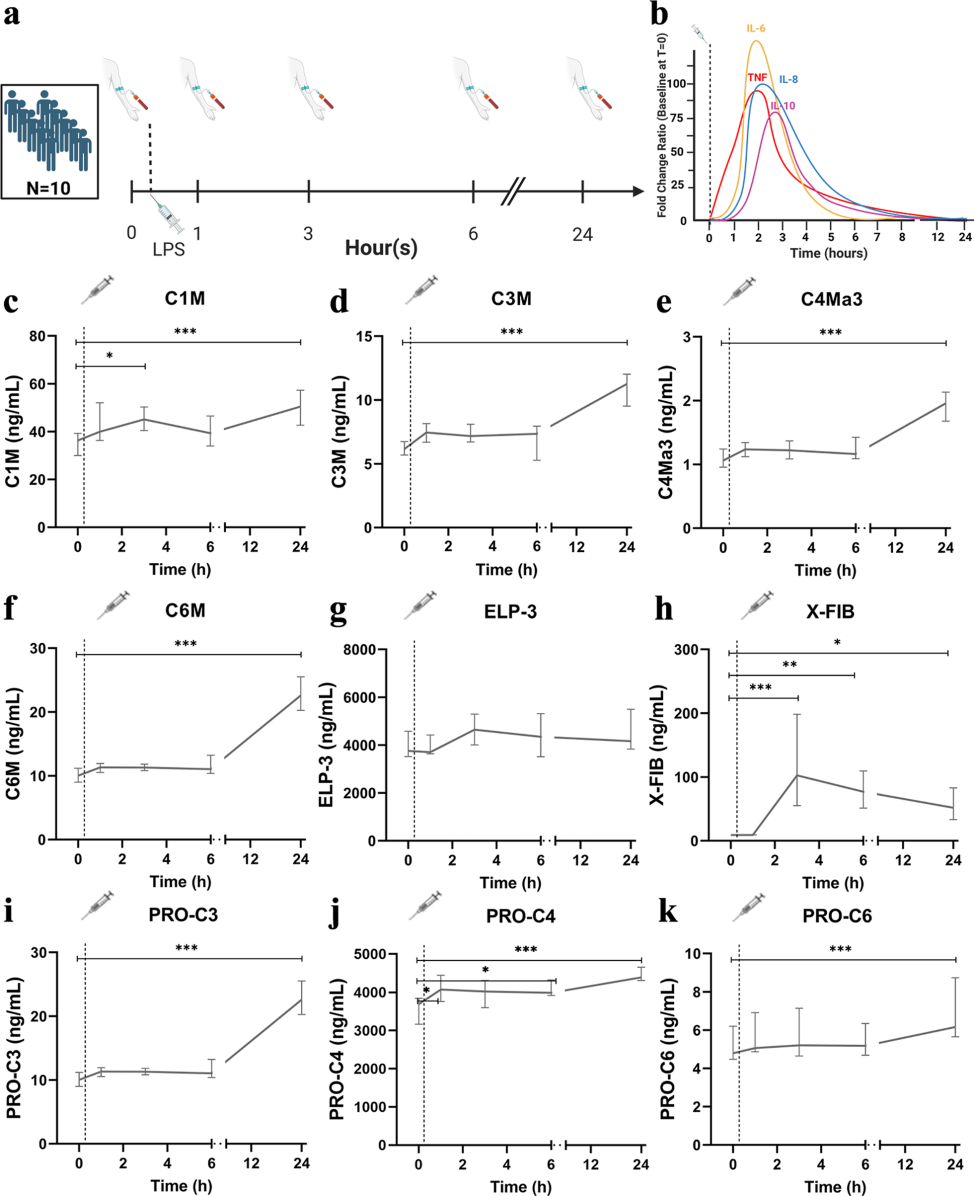
Human endotoxemia induces alterations in circulating ECM neo-epitopes. Schematic depiction of the experimental human endotoxemia model (**a**) and the induced inflammatory response (**b**), derived from previously published data [15] Plasma concentrations of C1M (**c**), C3M (**d**), C4Ma3 (**e**), C6M (**f**), ELP-3 (**g**), X-FIB (**h**), and PRO-C3 (**i**), PRO-C4 (**j**) and PRO-C6 (**k**) were measured in 10 healthy male individuals at baseline (T0) and at 1 h, 3 h, 6 h and 24 h after LPS administration. The dotted line indicates the moment of LPS administration (immediately after T0). Data are presented as median and interquartile range. *p < 0.05; **p < 0.01; ***p < 0.001 compared with T0, calculated by Friedman test followed by post-hoc testing with Bonferroni correction. *IL-6* Interleukin-6, *IL-8* Interleukin-8, *IL-10* Interleukin-10, *TNF* tumor necrosis factor, *LPS* lipopolysaccharide. Figure 1a and 1b were created with BioRender.com

Data and samples from the human endotoxemia experiments were obtained from 10 healthy male volunteers constituting the (normoxic) control arm of a study investigating the effects of hypoxia and hyperoxia on systemic inflammation (ClinicalTrials.gov identifier NCT01978158) [15, 16]. Endotoxemia was induced by intravenous administration of 2 ng/kg purified LPS (US Standard Reference Endotoxin Escherichia Coli O:113) obtained from the Pharmaceutical Development Section of the National Institutes of Health (Bethesda, MD, USA). Approval for the study was obtained from the local ethics committee of the Radboud university medical center (registration no. 2013-290), all subjects provided written informed consent, and all experiments were performed in accordance with the Declaration of Helsinki. The study procedures are described in detail elsewhere [15, 16] and graphically depicted in Fig. 1a. The experimental human endotoxemia model is characterized by a profound but relatively short-lived systemic inflammatory response, illustrated by increased plasma levels of pro-inflammatory cytokines TNF, IL-6 and IL-8 (peaking at 2 h following administration) and the anti-inflammatory cytokine IL-10 (peaking at 3 h post-LPS, Fig. 1b), as published previously [15].

### Septic shock patients

In the context of a prospective observational cohort study, lithium-heparin anticoagulated plasma samples from 43 patients with new-onset septic shock admitted to the ICU of Radboud university medical center between March 2012 and June 2015 were randomly selected based on sample availability (Table 1). Septic shock was defined as refractory hypotension that requires norepinephrine administration less than 24 h after ICU admission [17, 18]. Sepsis was defined in accordance with the SCCM/ESICM/ACCP/ATS/SIS International Sepsis Definitions Conference [18]. Patients without an arterial line were excluded. Exclusion criteria were chronic bowel disease, immune suppression, and documented chronic renal disease (serum creatinine concentration > 150 μmol/L). The study was carried out in the Netherlands in accordance with the applicable rules concerning the review of research ethics committees and informed consent. This was acknowledged by the local ethics committee of the Radboud university medical center (registration no. 2016-2923). All patients or legal representatives were informed about the study details and could refuse to participate. Except for C1M, PRO-C4, and ELP-3, data of the 10 experimental human endotoxemia subjects obtained at timepoint T0 (before LPS administration) were used as a healthy control comparison group for the septic shock patients. Furthermore, we added concentrations of healthy controls obtained from previously published literature as dotted lines in the graphs. Age and sex of these controls were more comparable to those of the septic shock patients included in our study [19–23].

**Table 1:** Characteristics of patients with septic shock

	Septic shock patients (N = 43)
Sex (male %)	25 (58.1%)
Age (years)	67.0 [59.0–77.0]
BMI (kg/m^2^)	25.0 [23.1–29.1]
Apache II score	22.0 [19.0–29.0]
Sepsis source	
Pulmonary (%)^a^	21 (48.8%)
Non-pulmonary (%)^a^	25 (58.1%)
Comorbidities	
Cardiovascular diseases (%)	23 (53.5%)
Pulmonary diseases (%)	17 (39.5%)
Oncological diseases (%)	19 (44.2%)
Immune compromised (%)	11 (25.6%)
Laboratory examination	
Leukocytes	14.6 (9.0–19.7)
IL-6	243.1 (44.3–930.0)
Mechanical ventilation (%)	43 (100%)
AKI requiring dialysis (%)	14 (32.6%)
Length of ICU stay (days)	12.0 [7.0–20.0]
Length of hospitalization (days)	30.0 (18.0–47.0)
ICU mortality (%)	7 (16.3%)
90-day mortality (%)	16 (37.2%)

Data are presented as percentage or median [IQR]

^a^Percent of total identified potential sepsis sources; some patients had multiple sources

### Measurements of plasma cytokine concentrations

In the endotoxemia experiments, EDTA-anticoagulated blood samples were collected at set time points (T = 0, 1, 1.5, 2, 2.5, 3, 4, 6, 8, and 24 h) after LPS administration. In septic shock patients, EDTA-anticoagulated blood samples were collected within 24 h of ICU admission. All samples were immediately centrifuged upon withdrawal (2000 g, 10 min, 4 °C), after which plasma was stored at − 80 °C until further analysis. Concentrations of TNF, IL-6, IL-8, and IL-10 were measured in a single batch using a Luminex assay (detection range: 3.2–10,000 pg/mL) following the manufacturer’s instructions (Milliplex, Millipore, Billerica, MA, USA).

### Measurements of circulating ECM neo-epitopes

In the endotoxemia experiments, ECM neo-epitopes were measured in lithium-heparin anticoagulated plasma samples that were collected immediately before LPS administration (designated as timepoint T0) and at T 1 h, 3 h, 6 h and 24 h thereafter. Blood samples were centrifuged upon withdrawal (2000 g, 10 min, 4 °C), after which plasma was stored at − 80 °C. In septic shock patients, lithium-heparin anticoagulated plasma samples were collected within 24 h of ICU admission and were centrifuged upon withdrawal (2000 g, 10 min, 4 °C), after which plasma was stored at − 80 °C. Neo-epitopes were measured using specific competitive enzyme-linked immunosorbent assays (ELISA) and competitive immunoassays on an automated platform (IDS i10) that employ neo-epitope-specific monoclonal antibodies developed by Nordic Bioscience (Herlev, Denmark). These assays were designed for the detection of specific MMP- mediated degradation fragments of type I collagen (C1M) [24], type III collagen (C3M) [25], type IV collagen alpha 3 chain (C4Ma3) [26] and type VI collagen alpha 1 chain (C6M) [27]. In addition, the ADAMTS-2 mediated release of N-terminal pro-peptide of type III collagen (PRO-C3) [28], an internal 7S domain of type IV collagen (P4NP 7S, PRO-C4) [29], C-terminal type Via3 collagen (PRO-C6, endotrophin) [30], a neo-epitope of plasmin-mediated degradation of cross-linked fibrin β-chain (D-dimer, X-FIB) [31], and a neo-epitope of proteinase-3 mediated degradation of elastin (ELP-3) [19] were measured. The assays were performed as previously described using the nordicC1M™, nordicC3M™, nordicC4Ma3™, nordicC6M™, nordicPRO-C3™, nordicPRO-C4™, nordicPRO-C6™, nordicX-FIB™, and nordicELP-3™ assays (Nordic Bioscience) [19, 24–31].

C1M, PRO-C4, and ELP-3 levels were measured using two different platforms for which actual levels measured were not comparable between the two cohorts (human endotoxemia model and septic shock patients). Therefore, as a control group for C1M and PRO-C4 measurements, data from thirty-three healthy donors were made available by Nordic Bioscience (N = 33 male 45%, age 36 [28–52] years). Lithium heparin plasma from healthy donors were obtained from the commercial vendor BioIVT (West Sussex, UK). For ELP-3, previously published control data made available by Nordic Bioscience was used (N = 98, male 50% age (mean) 59 years) [19]. This allowed for comparison of data from septic shock patients and control data measured on the same platform.

### Statistical analysis

Data are presented as percentage or median [interquartile range, IQR]. For the experimental human endotoxemia data, there were four missing measurements due to technical errors and low sample volume (three PRO-C4 measurements at T 24 h and one C4Ma3 measurement at T 6 h). Missing data were imputed using the average value of the other subjects for that specific biomarker and timepoint.

Longitudinal experimental human endotoxemia data were analysed using Friedman tests followed by post-hoc testing with Bonferroni correction, with T0 serving as the reference timepoint. Comparisons between healthy controls and septic patients were conducted using Mann–Whitney U tests. Correlations between inflammatory cytokines and ECM neo-epitopes were analysed using Spearman’s tests.

For all tests, a two-tailed *p* value of ≤ 0.05 was considered statistically significant. Graphing and calculations were performed using GraphPad Prism version 9.1.0 (GraphPad Software, Boston, Massachusetts, USA) and IBM SPSS Statistics for Windows (Version 28.0. Armonk, NY: IBM Corp), respectively.

## Results

### Human endotoxemia induces delayed occurrence of circulating ECM neo-epitopes

We hypothesized that release of ECM degradation neo-epitopes that were detectable in plasma may be triggered by systemic inflammation. To investigate this, we used a human model of systemic inflammation, induced by administration of LPS. We used samples from ten healthy male volunteers (age 21 [19–22] years, BMI 22.2 [20.5–24.2] kg/m^2^) who underwent experimental endotoxemia (experimental setup and plasma cytokine response depicted in Fig. 1a, b, respectively) to serially measure the levels of various ECM neo-epitopes.

Except for ELP-3, concentrations of all circulating degradation ECM neo-epitopes were increased at 24 h after LPS administration compared to baseline (T0): C1M: 48.3 [36.7–53.8] versus 34.2 [29.4–38.3] ng/mL *p* < 0.001, C3M: 10.7 [9.4–11.5] versus 6.6 [6.0–7.4] ng/mL *p* < 0.001, C4Ma3: 2.0 [1.7–2.2] versus 1.0 [1.0–1.2] ng/mL *p* < 0.001, C6M: 22.6 [18.5–24.5] versus 10.8 [9.4–12.1] ng/mL *p* < 0.001, and X-FIB: 51.7 [31.9–95.3] versus 9.1 [9.1–9.1] ng/mL *p* < 0.001 (Fig. 1c–h). Furthermore, concentrations of degradation neo-epitopes C1M and X-FIB were already increased at 3 h after LPS administration (41.5 [33.9–45.2] versus 34.2 [29.4–38.3] ng/mL *p* < 0.05 and 140.7 [65.2–282.8] versus 9.1 [9.1–9.1] ng/mL *p* < 0.001, respectively) (Fig. 1c, h). However, while C1M concentrations continued to rise over time, the concentration of X-FIB peaked at T 3 h and slowly decreased afterwards.

We also investigated whether ECM synthesis was altered by systemic inflammation. Similar to the degradation related ECM neo-epitopes, concentrations of ECM synthesis markers PRO-C3: 9.6 [7.2–11.9] versus 6.2 [5.3–6.9] ng/mL *p* < 0.001, PRO-C4: 4245.3 [3686.5–4386.2] versus 3648.2 [3267.7–3807.7] ng/mL *p* < 0.001, and PRO-C6: 6.3 [5.7–8.1] versus 4.7 [4.2–6.0] ng/mL *p* < 0.001 were increased 24 h after LPS administration compared with baseline (Fig. 1i–k). Furthermore, for PRO-C4, an early increase was also observed at 1 h after LPS administration compared with baseline (T0) (3867.1 [3501.4–4075.8] vs 3648.2 [3267.7–3807.7] ng/mL *p* < 0.05) (Fig. 1j).

Together, these data show that ECM degradation and synthesis is triggered in a human model of LPS-induced systemic inflammation. Interestingly, peak levels of most circulating ECM neo-epitopes are observed after resolution of the inflammatory response. However, not all circulating ECM neo-epitopes concentrations were altered, suggesting that systemic inflammation induces specific ECM alterations.

### Circulating ECM neo-epitopes are increased in septic shock patients

We next determined whether (similar) circulating alterations in ECM neo-epitopes were present in critically ill patients with new-onset septic shock (age 67.0 [59.0–77.0] years, APACHE II score 22.0 [19.0–29.0]). The characteristics of these patients are shown in Table 1.

**Fig. 2:**
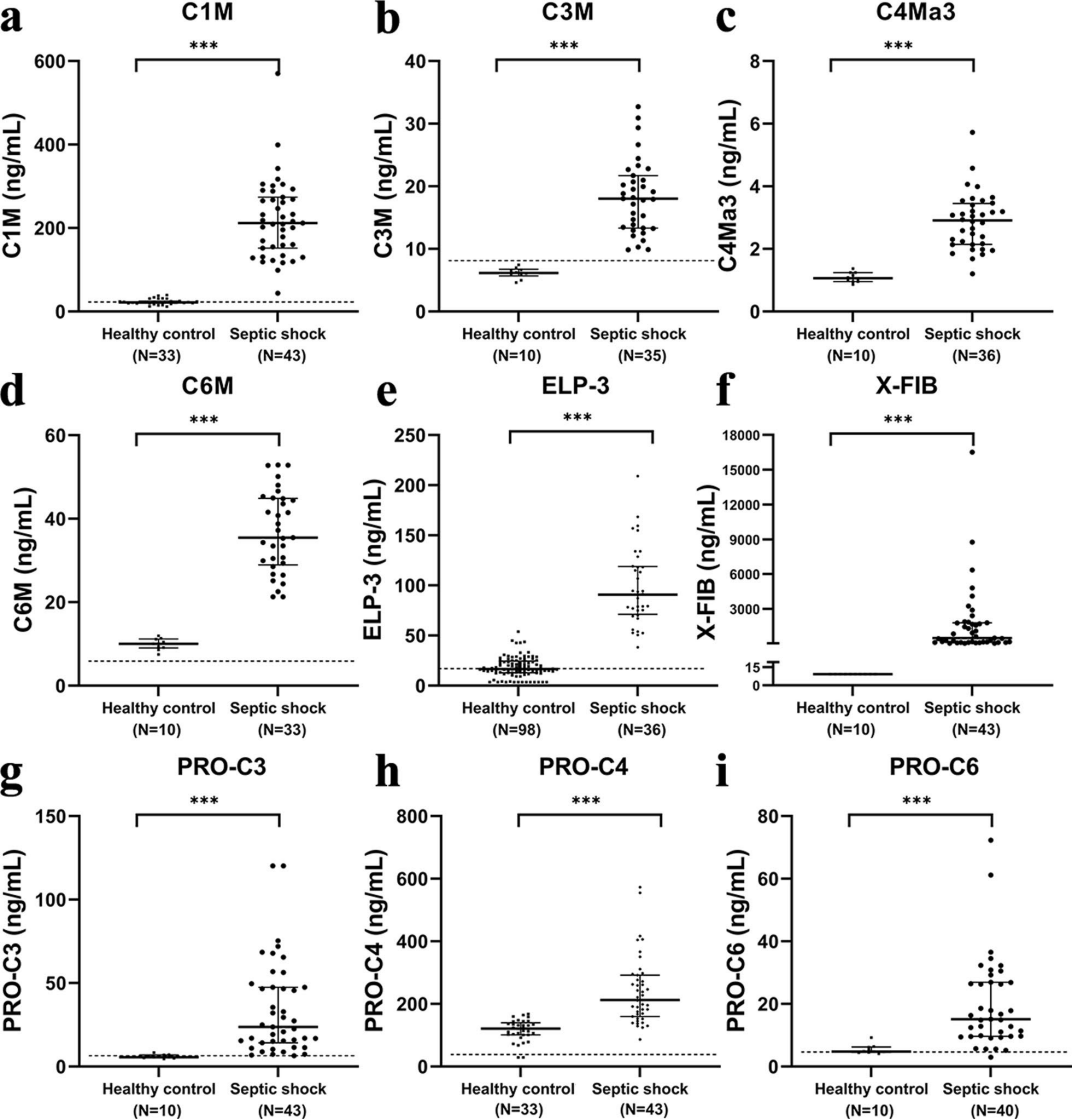
Patients with septic shock display elevated levels of circulating ECM neo-epitopes compared to healthy individuals. Plasma concentrations of C1M (**a**), C3M (**b**), C4Ma3 (**c**), C6M (**d**), ELP-3 (**e**), X-FIB (**f**), and PRO-C3 (**g**), PRO-C4 (**h**) and PRO-C6 (**i**) were measured in plasma samples collected at ICU admission from patients with septic shock (n = 43). Healthy control data of C3M, C4Ma3, C6M and X-FIB were obtained from the experimental human endotoxemia model at T0 (before LPS administration). Healthy control data for C1M and PRO-C4 levels were obtained from 33 healthy donors (in-house controls measured by Nordic Bioscience), and the levels of ELP-3 measured in control individuals were reported previously [19]. Each symbol represents a single individual or patient. The dotted lines represent the circulating levels in healthy individuals reported previously [19–23]. Data are presented as median and interquartile range. ***p < 0.001 calculated using Mann–Whitney U tests

Compared to healthy control subjects, the concentrations of all measured circulating ECM degradation neo-epitopes in septic shock patients were increased: C1M (21.9 [19.7–24.3] vs 211.6 [151.7–274.0] ng/mL *p* < 0.001), C3M (6.6 [6.0–7.4] vs 18.0 [13.3–21.7] ng/mL *p* < 0.001), C4Ma3 (1.0 [1.0–1.2] vs 2.9 [2.1–3.4] ng/mL *p* < 0.001), C6M (10.8 [9.4–12.1] vs 35.5 [28.9–44.9] ng/mL *p* < 0.001), ELP-3 (16.5 [12.8–24.1] vs 90.8 [71.1–118.9] ng/mL *p* < 0.001) and X-FIB (9.1 [9.1–9.1] vs 484.7 [101.4–1786.9] ng/mL *p* < 0.001) (Fig. 2a–f).

Similar to what was observed in the measurements of degradation neo-epitopes, concentrations of all ECM synthesis neo-epitopes were increased in patients with septic shock compared to healthy controls: PRO-C3 (6.2 [5.3–6.9] vs 23.6 [14.2–47.4] ng/mL *p* < 0.001), PRO-C4 (120.9 (101.0–139.8) vs 212.2 [159.7–291.6] ng/mL *p* < 0.001) and PRO-C6 (4.7 [4.2–6.0] vs 15.1 [9.7–26.9] ng/mL *p* < 0.001) (Fig. 2g–i).

#### Inflammation is related to ECM remodelling in patients with septic shock

To determine whether there was a relationship between systemic inflammation and ECM remodelling, we performed correlation analyses between cytokine concentrations and ECM neo-epitopes concentrations in patients with septic shock at ICU admission. Of note, the group size of the endotoxemia cohort was too small for this purpose. We identified several statistically significant correlations between cytokines (IL-10, IL-6, IL-8, and TNF) and circulating ECM neo-epitopes, albeit weak (Table 2 and Additional file 1, Fig. S1). TNF concentrations were correlated with 7 out of the 9 measured ECM neo-epitopes, encompassing both degradation and synthesis neo-epitopes. TNF, IL-8, and IL-10 were correlated with degradation neo-epitopes C3M, C4Ma3 and C6M. In addition, all pro-inflammatory cytokines correlated positively with X-FIB. As inflammation is known to be related to disease severity, we also explored the relationship between APACHE II severity scores and ECM neo-epitopes at ICU admission, but no significant correlations were found (Additional file 1, Fig. S2).

**Table 2:** Circulating levels of ECM neo-epitopes weakly correlate with plasma levels of inflammatory cytokines

ICU admission	IL-10		IL-6		IL-8		TNF	
	Correlation coefficient	*p* value	Correlation coefficient	*p* value	Correlation coefficient	*p* value	Correlation coefficient	*p* value
C1M	0.225	0.147	0.080	0.611	0.242	0.118	0.214	0.169
C3M	**0.344**	0.043	0.179	0.304	**0.348**	0.041	**0.492**	0.003
C4Ma3	**0.354**	0.034	0.230	0.176	**0.467**	0.004	**0.412**	0.013
C6M	**0.354**	0.044	0.171	0.340	**0.353**	0.044	**0.414**	0.017
ELP-3	0.291	0.085	0.080	0.644	0.270	0.111	**0.334**	0.046
X-FIB	0.273	0.077	**0.411**	0.006	**0.469**	0.002	**0.392**	0.009
PRO-C3	0.288	0.061	0.293	0.057	**0.505**	0.001	**0.494**	0.001
PRO-C4	0.201	0.196	0.076	0.630	0.157	0.315	0.259	0.094
PRO-C6	0.171	0.298	0.259	0.112	0.199	0.224	**0.335**	0.037

Spearman correlation (r and p) between concentrations of inflammatory cytokines and circulating ECM neo-epitopes in septic shock patients at ICU admission. Bold values signify correlations with a *p* value < 0.05

#### Increased concentrations of circulating C6M, ELP-3 and X-FIB are associated with mortality in patients with septic shock

**Fig. 3:**
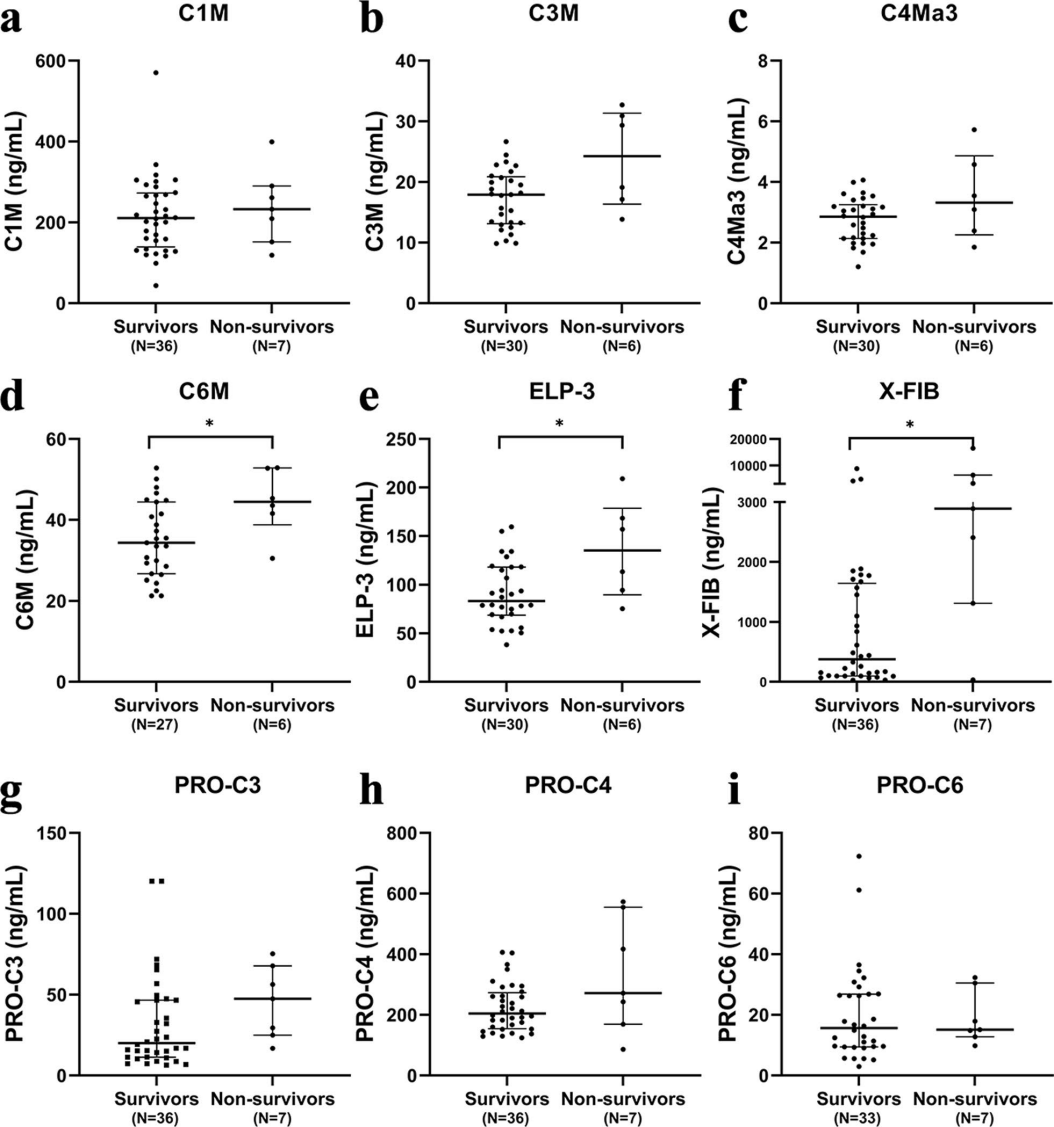
Circulating levels of C6M, ELP-3, X-FIB, and PRO-C3 at ICU admission are associated with mortality of patients with septic shock. Plasma concentrations of C1M (**a**), C3M (**b**), C4Ma3 (**c**), C6M (**d**), ELP-3 (**e**), X-FIB (**f**), and PRO-C3 (**g**), PRO-C4 (**h**) and PRO-C6 (**i**) were measured in plasma samples collected at ICU admission from patients with septic shock (n = 43). The patients were divided into two groups, ICU survivors (n = 36) and ICU non-survivors (n = 7). Each symbol represents single patient. Data are presented as median and interquartile range. *p < 0.05; *p* < 0.01; ***p < 0.001 calculated using Mann–Whitney U tests

To investigate whether the levels of circulating ECM neo-epitopes were related to patient outcome, we first determined whether the concentrations of ECM degradation neo-epitopes measured at ICU admission were associated with ICU mortality (Fig. 3). In total, 7 out of 43 patients included in this study died during their ICU stay. Plasma concentrations of C6M (44.5 [38.8–52.8] vs 34.3 [26.7–44.4] ng/mL *p* < 0.05), ELP-3 (135.1 [89.7–178.6] vs 83.2 [68.7–118.1] ng/mL *p* < 0.05) and X-FIB (2891.0 [1313.0–6345.4] vs 377.4 [97.4–1647.1] ng/mL *p* < 0.05) were higher in septic shock patients that died in the ICU than in those who survived their ICU stay (Fig. 3a–f).

In contrast, concentrations of ECM synthesis neo-epitopes in patients who died were comparable to those observed in patients who survived, PRO-C3 (47.4 [24.9–67.8] vs 20.0 [11.3–46.5] ng/mL *p* = 0.065) and PRO-C4 (271.8 [168.8–5540.8] vs 204.7 [154.6–273.0] ng/mL *p* = 0.157), PRO-C6 (15.1 [12.8–30.5] vs 15.6 [9.4–26.9] ng/mL *p* = 0.534 (Fig. 3g–i). These results suggest that ECM degradation and in particular alterations leading to increased plasma levels of C6M, ELP-3, and X-FIB are associated with mortality in patients with septic shock.

## Discussion

In this exploratory study, we demonstrate that LPS administration in healthy volunteers induces ECM turnover. Furthermore, ECM turnover was also elevated in patients with septic shock and related to the extent of inflammation. Finally, ECM degradation, as measured by C6M, ELP-3 and X-FIB on ICU admission is associated with mortality in these patients, whereas ECM synthesis is not. These data indicate that acute systemic inflammation induces ECM turnover and that circulating ECM neo-epitopes may have a role as biomarkers in septic shock.

Systemic inflammation is an early feature of patients with septic shock which causes multiple organ alterations that may lead to poor acute and long-term patient outcomes. To investigate the intrinsic role of inflammation, so without organ failure, comorbidities, medication etc. on ECM turnover, we made use of the experimental human endotoxemia model. In this highly controlled and reproducible model of systemic inflammation, robust indications of increased ECM turnover were observed 24 h after LPS administration.

Our results on circulating ECM neo-epitopes during human endotoxemia and in patients with septic shock generate key insights into ECM biology. Firstly, ECM degradation (C1M, C3M, C4Ma3, C6M, ELP-3, X-FIB) and synthesis (PRO-C3, -C4, -C6) appear to occur simultaneously during human endotoxemia. Secondly, ECM turnover in patients with septic shock is related, albeit to a relatively limited extent, to the inflammatory response, which is known to be associated with disease severity. In accordance, we also demonstrated higher concentrations of degradation neo-epitopes ELP-3, C6M, and X-FIB in patients who did not survive. These degradation neo-epitopes of elastin and collagen type VI might reflect tissue damage of lung, vasculature, and kidney already at ICU admission [32–34]. The finding that APACHE II severity scores did not significantly correlate with any of the neo-epitopes may be attributed to the small sample size and comparatively low specificity of the score. Strikingly, neo-epitopes of collagen synthesis, PRO-C3, -C4, -C6, were not associated with mortality. It is tempting to speculate that this might reflect a tissue imbalance towards ECM degradation, thus contributing to decreased organ recovery.

The panel of ECM neo-epitopes measured was based on the coupling between degradation and synthesis neo-epitopes and the native location of the ECM parent molecules in the tissues. X-FIB, similar to D-dimer, is a degradation product of fibrin, which is mainly present within the circulation during coagulation activity and released upon clot resolution [35]. Collagen type VI (C6M and PRO-C6) is a microfibrillar beaded collagen connects the basement membrane and interstitial matrix in all tissues [36, 37]. Collagen type IV (C4Ma3 and PRO-C4) is located within the basement membrane of which the alpha3 chain is mainly found in the alveolar basement membrane [29], and produced by endothelial and epithelial cells [38, 39]. Collagen type I (C1M and PRO-C1) and type III (C3M and PRO-C3) are major constituents of the interstitial matrix in various organs [40]. ELP-3 is a fragment generated by proteinase-3 (a neutrophil degranulation product) mediated degradation of elastin, which is present in the lung [35]. The anatomical location of the ECM, and therefore the susceptibility for degradation, might be reflected in the neo-epitope kinetics during human endotoxemia. Release of most of the collagen neo-epitopes showed a somewhat delayed responses to LPS administration, only appearing after 24 h. However, PRO-C4 was already elevated within 1 h post-LPS challenge. This might be due to its location in the vascular basement membrane, which is anatomically close to the vascular compartment in which LPS was administered. This may also explain the rapid increase of X-FIB after LPS administration, as this is a degradation product of fibrin which is present within the circulation [41, 42].

Our findings in critically ill patients are partially in line with previous studies in sepsis and ARDS [11, 12, 43], although these mainly focussed on collagen type I and III. In sepsis, fragments of collagen type I degradation and collagen type I and III synthesis were shown to be elevated [11, 43]. Our data show the potential of exploring a larger panel of ECM neo-epitopes in sepsis to provide insights into the pathophysiology of organ failure and prognostication of possible organ recovery. Not only can inflammation lead to aberrant ECM turnover, in turn causing tissue remodelling, this aberrant remodelling itself could contribute to a pro-inflammatory tissue environment. This is mediated through ECM fragments and matricellular proteins capable of facilitating immune cell migration, activation, and persistent inflammation, thereby impairing organ recovery [44] and creating a vicious circle detrimental to the patient. In addition, at a macroscopic level, aberrant tissue remodelling (resulting in fibrosis) can structurally impair tissues such as the lung, resulting in failure to wean from mechanical ventilation, excessive fibroproliferation and ultimately mortality [12]. The ECM neo-epitopes identified in this study, which are detectable in the circulation, could provide information on ongoing tissue damage and ECM turnover even after inflammation has partially resolved. In addition, failure of ECM synthesis could signal impairment of organ recovery. These hypotheses should be tested in larger patient cohorts, preferably using longitudinal sampling, and endpoints of organ recovery should be available in these datasets.

A strength of this study is the inclusion of both healthy volunteers subjected to a controlled model of inflammation and patients with septic shock, which led to the novel finding that acute systemic inflammation induces ECM turnover. However, our study also has several limitations. First, the sample size was relatively small, especially for the human endotoxemia data. Second, we did not include age, sex-, and co-morbidity-matched controls. Nevertheless, the ECM component concentrations in septic shock patients observed in our study are several folds higher than those of the young healthy male controls from the human endotoxemia study, which is unlikely to be solely the result of differences in age, sex, and/or comorbidities [10]. Furthermore, to partially address the lack of matched controls, we also included previously published neoepitope concentrations of healthy individuals with a similar age and sex distribution [19–23]. These concentrations were very similar to those observed in our young healthy male volunteers, or in case of C1M and PRO-C4, to the in-house controls provided by the neoepitope ELISA manufacturer. Third, the samples from our sepsis cohort were relatively old compared with those obtained from healthy controls. This may have influenced the measured concentrations of neoepitopes, for instance through increased degradation in older samples with time. However, if this is the case, actual concentrations of neo-epitopes would be higher in septic shock patients, not lower, merely resulting in an underestimation of the real difference between these patients and healthy controls and not a false-positive finding. Fourth, we only included males in the endotoxemia experiments, because we wanted our study population to be as homogenous as possible. There are considerable differences in the cytokine response to LPS between males and females [45]. This is likely influenced by menstrual cycle-related hormonal variations resulting in greater interindividual variation between females. As human endotoxemia experiments are very labour-intensive and expensive studies, and for ethical reasons (we want to expose as few volunteers as possible to endotoxemia), we only include male subjects. However, there are no known or theoretical reasons why ECM turnover would be different in females, and both sexes were represented in the septic patient cohort. Finally, we measured a specific selection of ECM neo-epitopes, chosen based on their tissue location. This selection is not exhaustive, although the number of measured ECM neo-epitopes is larger than in any of the previously published studies performed in critically ill patients to date.

## Conclusions

Our study provides valuable insights into ECM turnover and tissue remodelling dynamics during systemic inflammation and septic shock. We identified ECM neo-epitopes which could provide novel insight into pathophysiology of organ failure, recovery, and prognostication of critically ill patients.

### Supplementary Information

**Additional file 1**: **Supplementary data 1.** Correlation between cytokines and neo-epitopes in septic shock patients. **Figure S1.1.** Correlation between IL-10 and neo-epitopes. **Figure S1.2.** Correlation between IL-6 and neo-epitopes. **Figure S1.3.** Correlation between IL-8 and neo-epitopes. **Figure S1.4.** Correlation between TNF and neo-epitopes. **Supplementary data 2.** Correlation between disease severity (APACHE II) and neo-epitopes in septic shock patients. **Figure S2.** Correlation between disease severity (APACHE II) and neo-epitopes.

## Data Availability

Data is available upon reasonable request from the corresponding author.

## Abbreviations

ARDS: Acute respiratory distress syndrome
ECM: Extracellular matrix
LPS: Lipopolysaccharide
MMP: Matrix metalloproteinase
C1M: MMP degraded collagen type I
C3M: MMP degraded collagen type III
C4M: MMP degraded collagen type IV
C6M: MMP degraded collagen type VI
ELP-3: Proteinase-3 degraded elastin
X-FIB: Plasmin-degraded cross-linked fibrin
PRO-C3: Disintegrin and metalloproteinase with thrombospondin motifs 2 (ADAMTS-2) mediated release of N-terminal pro-peptide of type III collagen
PRO-C4: N-terminal pro-peptide of type IV collagen
PRO-C6: C-terminal pro-peptide of type VI collagen
ELISA: Enzyme-linked immunosorbent assays
IL-6: Interleukin-6
IL-8: Interleukin-8
IL-10: Interleukin-10
TNF: Tumour necrosis factor
