# Supplementary material for: Neo-epitope detection identifies extracellular matrix turnover in systemic inflammation and sepsis: an exploratory study
Source: Crit Care. 2024 Apr 12;28:120. doi: 10.1186/s13054-024-04904-4 (PMC11010428; doi:10.1186/s13054-024-04904-4)
Supplement: Supplementary file 1 — Additional file 1: Supplementary data 1. Correlation between cytokines and neo-epitopes in septic shock patients. Figure S1.1. Correlation between IL-10 and neo-epitopes. Figure S1.2. Correlation between IL-6 and neo-epitopes. Figure S1.3. Correlation between IL-8 and neo-epitopes. Figure S1.4. Correlation between TNF and neo-epitopes. Supplementary data 2. Correlation between disease severity (APACHE II) and neo-epitopes in septic shock patients. Figure S2. Correlation between disease severity (APACHE II) and neo-epitopes. [file 13054_2024_4904_MOESM1_ESM.docx]

**Supplementary file**

**Neo-epitope detection identifies extracellular matrix turnover in systemic inflammation and sepsis**

YiWen Fan^1,2,3^, Jill Moser^2^, Matijs van Meurs^2^, Dorien Kiers^4^, Jannie Marie Bülow Sand^5^, Diana Julie Leeming^5^, Peter Pickkers^4,6^, Janette K. Burgess^1,3^, Matthijs Kox^4,6,*^, Janesh Pillay^2,3,*^

*1 University of Groningen, University Medical Center Groningen, Department of Pathology and Medical Biology, Groningen, the Netherlands*

*2 University of Groningen, University Medical Center Groningen, Department of Critical Care, Groningen, the Netherlands.*

*3 University of Groningen, University Medical Center Groningen, Research Institute for Asthma and COPD, Groningen, the Netherlands.*

*4 Radboud university medical center, Department of Intensive Care Medicine, Nijmegen, the Netherlands.*

*5* Nordic Bioscience, Hepatic and Pulmonary Research, Herlev, Denmark*.*

*6 Radboud university medical center, Radboud Centre for Infectious Diseases (RCI), Nijmegen, the Netherlands.*

**Equal contribution*

**Supplementary data Appendix**

**Contents**

**Supplementary data 1: Correlation between cytokines and neo-epitopes in septic shock patients…………………………….….….….….….….….….….….….….….….….….….….….….….….…..…3**

Figure S1.1 Correlation between IL-10 and neo-epitopes………………..………………..3

Figure S1.2 Correlation between IL-6 and neo-epitopes………………………………….…4

Figure S1.3 Correlation between IL-8 and neo-epitopes………………………………....…5

Figure S1.4 Correlation between TNF and neo-epitopes..……………………………....…6

**Supplementary data 2: Correlation between disease severity (APACHE II) and neo-epitopes in septic shock patients.………………....…...……………………………....…...………………………......…7**

**Supplementary data 1: Correlation between cytokines and neo-epitopes in septic shock patients**


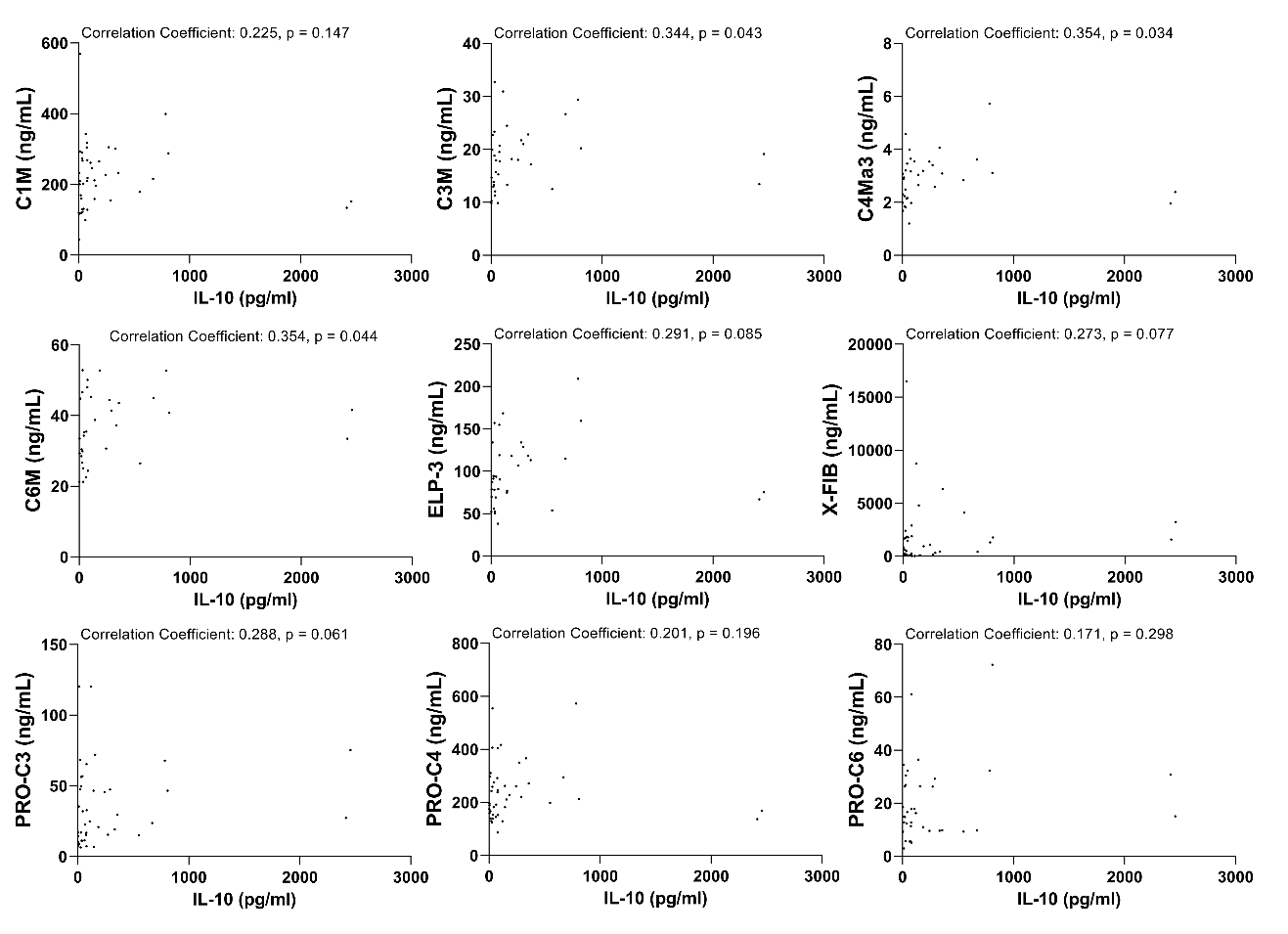


**Figure S1.1 Correlation between IL-10 and neo-epitopes**

Spearman correlation (r and p) between concentrations of IL-10 and circulating ECM neo-epitopes in septic shock patients at ICU admission.


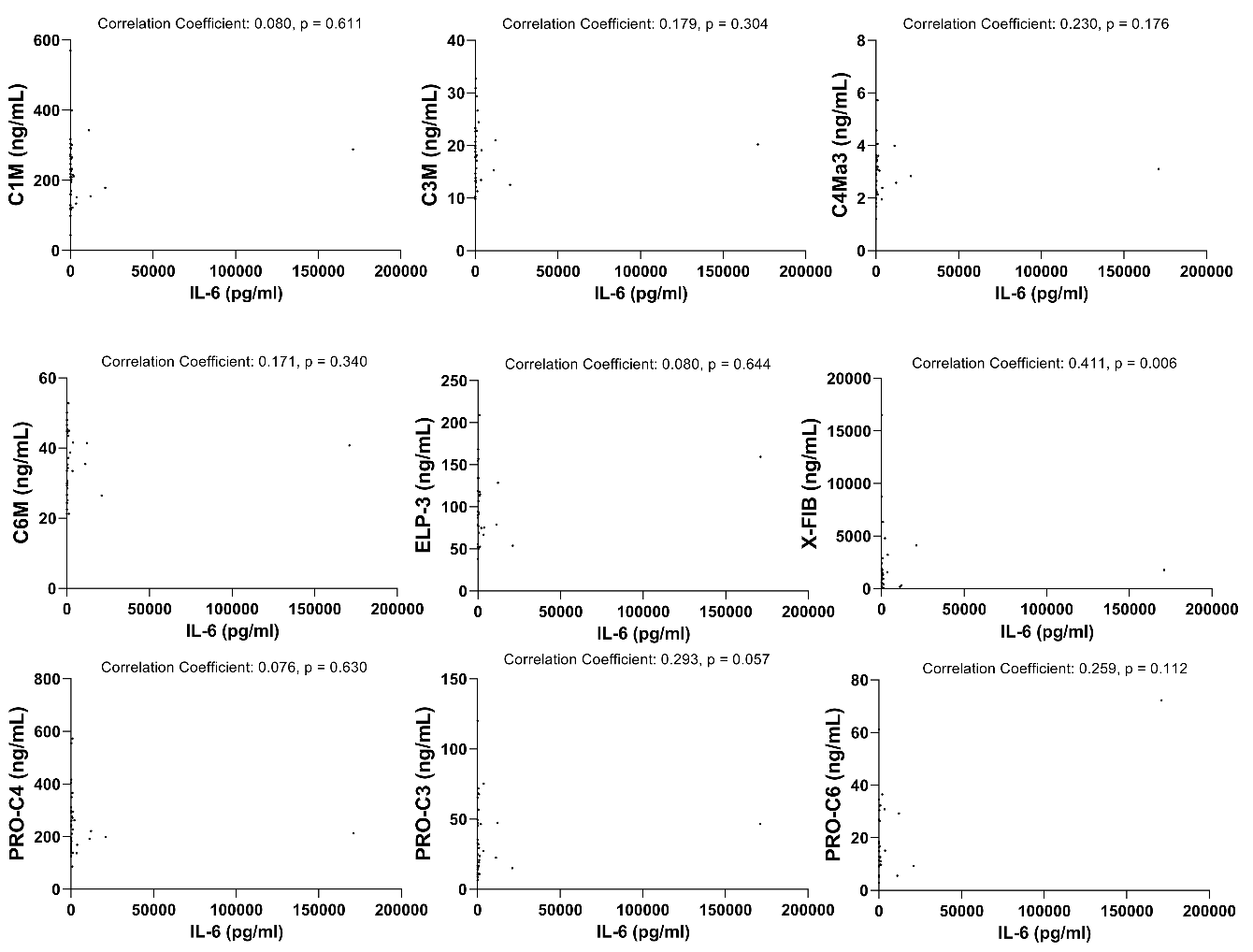


**Figure S1.2 Correlation between IL-6 and neo-epitopes**

Spearman correlation (r and p) between concentrations of IL-6 and circulating ECM neo-epitopes in septic shock patients at ICU admission.

**
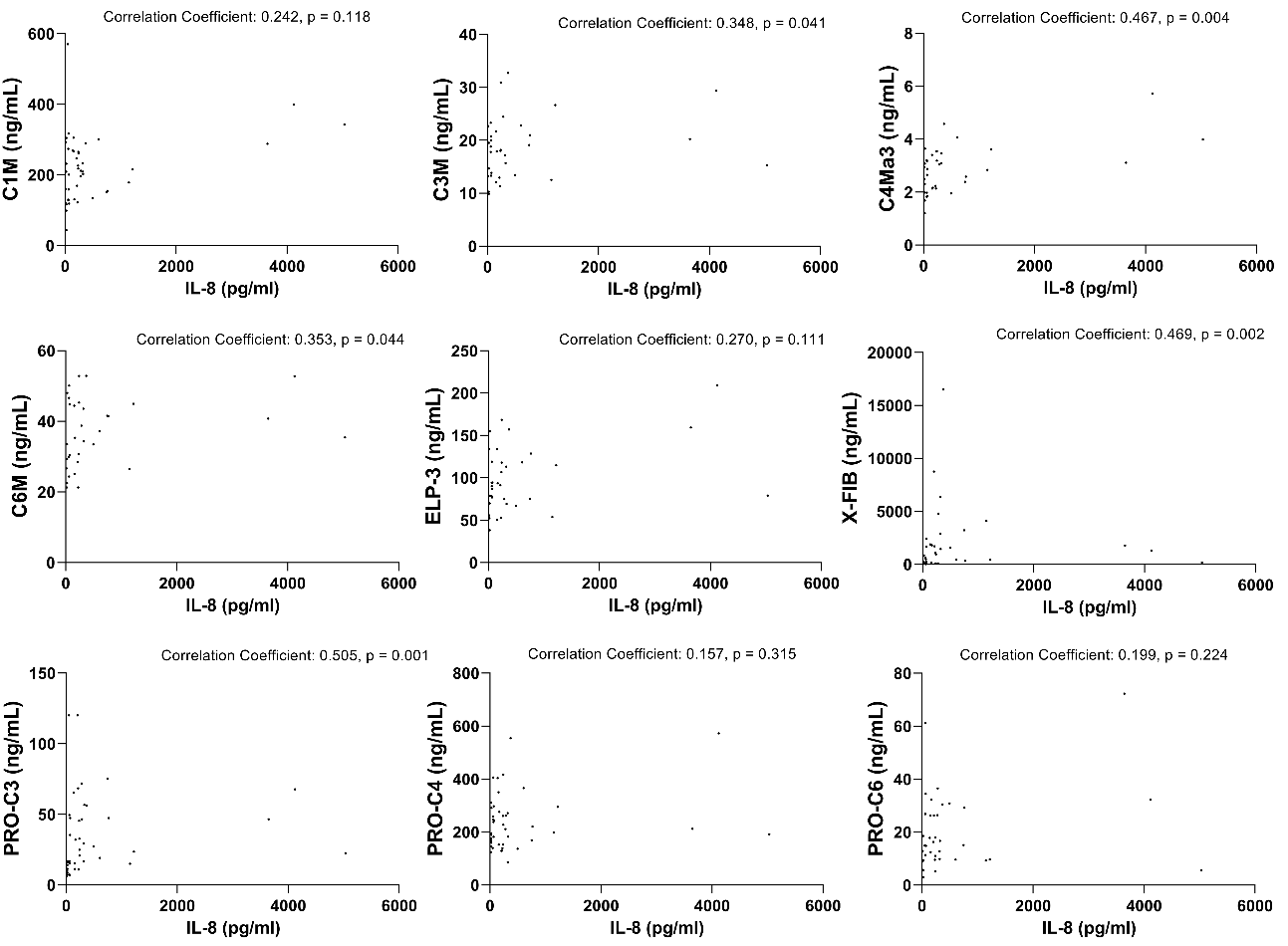
**

**Figure S1.3 Correlation between IL-8 and neo-epitopes**

Spearman correlation (r and p) between concentrations of Il-8 and circulating ECM neo-epitopes in septic shock patients at ICU admission.


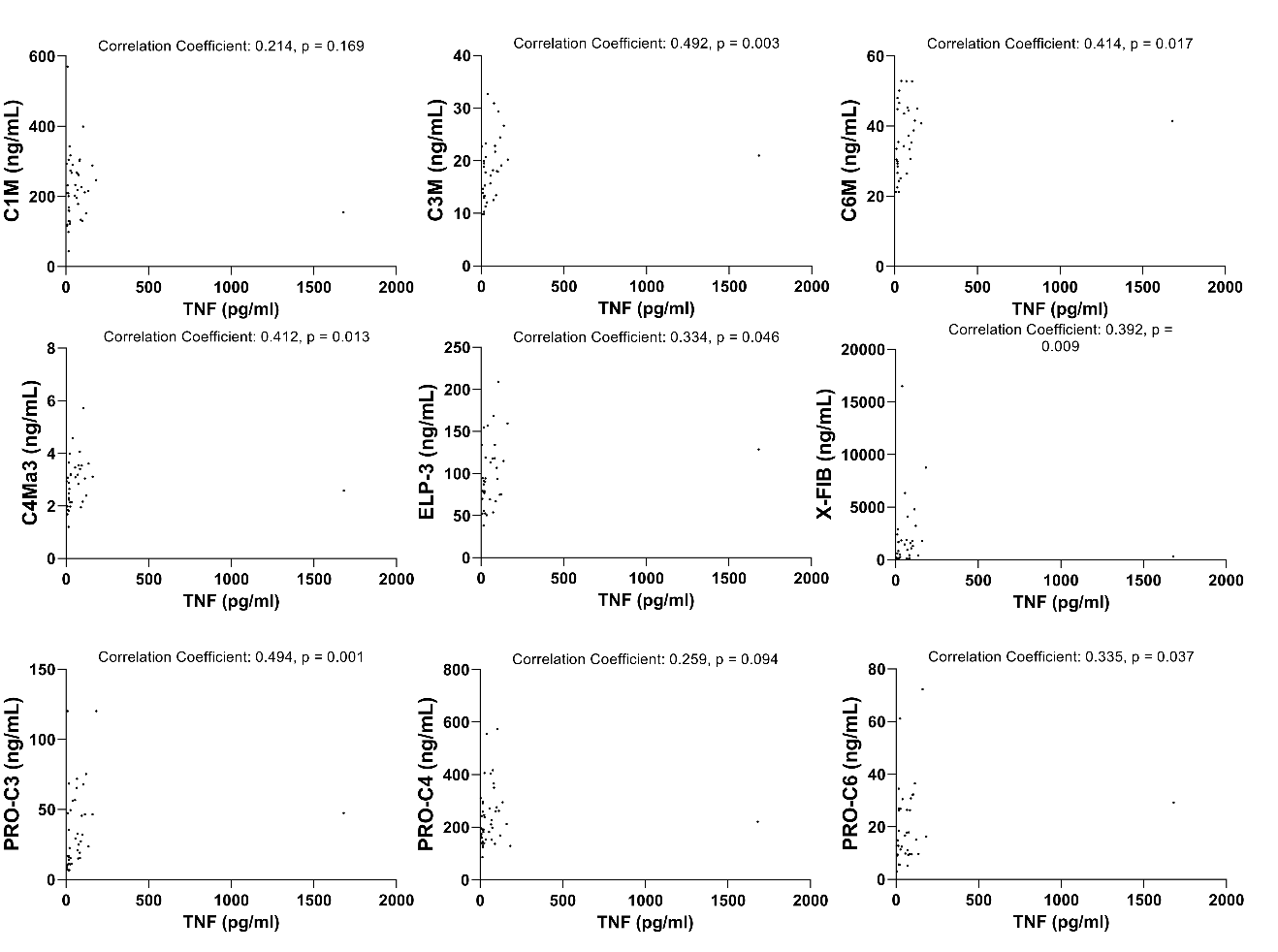


**Figure S1.4 Correlation between TNF and neo-epitopes**

Spearman correlation (r and p) between concentrations of TNF and circulating ECM neo-epitopes in septic shock patients at ICU admission.

**Supplementary data 2: Correlation between disease severity (APACHE II) and neo-epitopes**

**
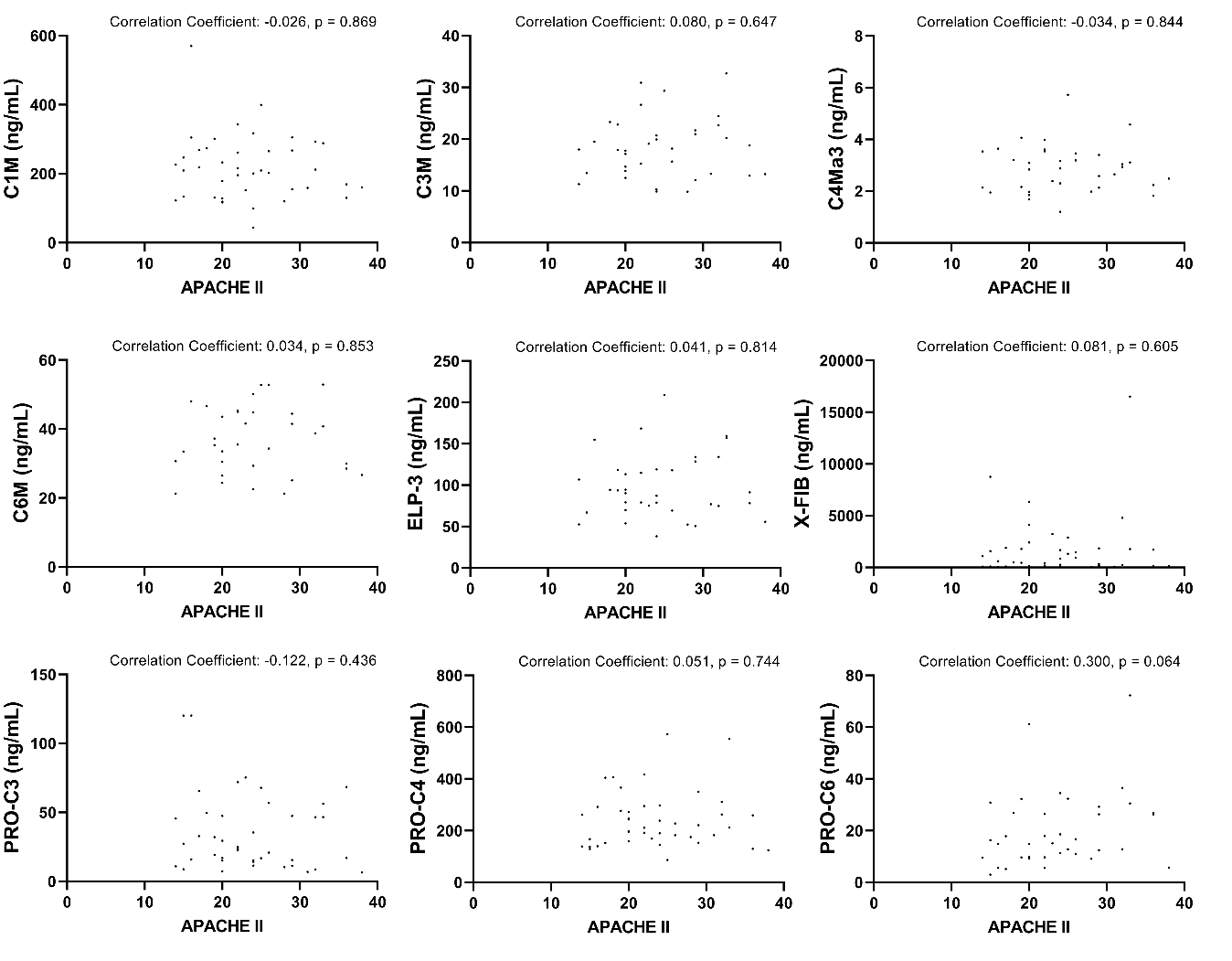
**

**Figure S2 Correlation between disease severity (APACHE II) and neo-epitopes**

Spearman correlation (r and p) between APACHE II scores and circulating ECM neo-epitopes in septic shock patients at ICU admission.
